# Physician-delivered motivational interviewing to improve adherence and retention in care among challenging HIV-infected patients in Argentina (COPA2): study protocol for a cluster randomized controlled trial

**DOI:** 10.1186/s13063-018-2758-5

**Published:** 2018-07-24

**Authors:** Omar Sued, Isabel Cassetti, Diego Cecchini, Pedro Cahn, Lina Bofill de Murillo, Stephen M. Weiss, Lissa N. Mandell, Manasi Soni, Deborah L. Jones

**Affiliations:** 1 Fundación Huésped, Pasaje Angel Peluffo 3932, C1202ABB Buenos Aires, Argentina; 2 Helios Salud, Peru 1515, Buenos Aires, Argentina; 3 Department of Psychiatry and Behavioral Sciences, University of Miami Miller School of Medicine, 1400 NW 10th Ave, Miami, FL USA

**Keywords:** Adherence, Retention, HIV, Motivational interviewing, Argentina, Physicians

## Abstract

**Background:**

“Challenging” HIV-infected patients, those not retained in treatment, represent a critical focus for positive prevention, as linkage to care, early initiation of antiretroviral therapy, adherence and retention in treatment facilitate viral suppression, thus optimizing health and reducing HIV transmission. Argentina was one of the first Latin American countries to guarantee HIV prevention, diagnosis and comprehensive care services, including antiretroviral medication, which removed cost and access as barriers. Yet, dropout occurs at every stage of the HIV continuum. An estimated 110,000 individuals are HIV-infected in Argentina; of these, 70% have been diagnosed and 54% were linked to care. However, only 36% have achieved viral suppression and 31% of those diagnosed delayed entry to care. To achieve meaningful reductions in HIV infection at the community level, innovative strategies must be developed to re-engage patients. Motivational Interviewing (MI) is a patient-centered approach and has been used by therapists in Central and South America to enhance motivation and commitment in substance use and risk reduction. Our pilot feasibility study utilized culturally tailored MI in physicians to target patients not retained in treatment in public and private clinics in Buenos Aires, Argentina. Results demonstrated that a physician-based MI intervention was feasible and effective in enhanced and sustained patient adherence, viral suppression, and patient-physician communication and attitudes about treatment among these patients at 6 and 9 months post baseline.

**Methods/design:**

This clinical trial seeks to extend these findings in public and private clinics in four urban population centers in Argentina, in which clinics (*n* = 6 clinics, six MDs per clinic site) are randomized to experimental (physician MI Intervention) (*n* = 3) or control (physician Standard of Care) (*n* = 3) conditions in a 3:3 ratio. Using a cluster randomized clinical trial design, the study will test the effectiveness of a physician-based MI intervention to improve and sustain retention, adherence, persistence, and viral suppression among “challenging” patients (*n* = 420) over 24 months.

**Discussion:**

Results are anticipated to have significant public health implications for the implementation of MI to re-engage and retain patients in HIV treatment and care and improve viral suppression through high levels of medication adherence.

**Trial registration:**

ClinicalTrials.gov, ID: NCT02846350. Registered on 1 July 2016.

**Electronic supplementary material:**

The online version of this article (10.1186/s13063-018-2758-5) contains supplementary material, which is available to authorized users.

## Background

“Challenging” patients, those not retained in treatment, represent a critical focus for effective HIV prevention efforts to achieve reduction in overall individual- and community-level viral burden [1, 2]. These “challenging” patients, here defined as those not retained in care, are often characterized by a lack of medication persistence, defined as obtaining treatment followed by a gap in care [3], inconsistent treatment visits, and repeated missed appointments. Such behavior renders antiretroviral therapy (ART) ineffective and typically results in treatment failure.

Argentina was one of the first Latin American countries to guarantee HIV comprehensive care services, which removed cost and access as barriers to care for HIV-infected patients. Yet, as in the USA, dropout occurs at every stage of the HIV continuum from linkage to retention, prescription and viral suppression. Of the estimated 110,000 HIV-infected individuals in Argentina, 70% have been diagnosed and 54% of those eligible (CD4 count < 500 cells/mm^3^) were linked to treatment. However, only 36% achieved viral suppression and 31% of those diagnosed delayed entry to care [4, 5]. Patients disengaged from care fail to achieve and maintain the benefits of antiretroviral therapy, such as optimal health and reduced potential for HIV transmission [2].

Ongoing “retention in care” is defined as attending two or more appropriately spaced visits within 1 year with an HIV medical provider [6]. If patients are not retained in care, rapid re-engagement and retention are essential, as achieving viral suppression requires twice as long with suboptimal retention [7]. The Argentina standard of care recommends patients re-engaged in care are evaluated quarterly for viral suppression, or more frequently as needed, for 1-year following re-linkage; thereafter, if immunologically stable and virologically suppressed, assessments are biannual. ART prescription refills are almost exclusively provided monthly by physicians; 2-month prescriptions are only provided for special circumstances or for private patients with insurer permission. Theoretically, retention can be enhanced through monitoring and tracking missed appointments, adapting clinic structures and healthcare systems to respond to barriers to care, and providing supportive information, skills and motivation to patients to enable them to navigate the health care system.

Our pilot study, Conexiones y Opciones Positivas en la Argentina (COPA), estimated that retention in care in urban clinics with high HIV rates ranged from 65 to 90%. Given universal access to care, patients with uncontrolled HIV constitute a significant public health threat due to an increased likelihood of viral resistance [8], treatment failure and HIV transmission; in fact, in 2013, 23.8% of those on medication were already on second-line ART [9]. Clearly, retention in care is a growing challenge to effective HIV treatment. This study addresses re-engagement of patients not retained in care following ART prescription.

Effective strategies to re-engage “challenging” patients must be developed and implemented if we are to achieve meaningful population-level decreases in HIV incidence. Our feasibility study targeting challenging patients was culturally tailored to the local setting and found to be both feasible and highly acceptable to patients, physicians, and clinic staff in public and private clinics in Buenos Aires, Argentina. The study identified barriers in public and private settings that were changed (e.g., systems to reschedule appointments and obtain prescriptions when medical appointments are missed, proximity of laboratory and pharmacy services), but while infrastructure development can facilitate engagement, ultimately patient-centered care relies on the physician-patient relationship [10].

Both physicians and patients were enthusiastic regarding the use of Motivational Interviewing (MI) during clinical consultations, and reported greater patient engagement in treatment. MI is a patient-centered approach and has been used by therapists in Central and South America to enhance motivation and commitment in substance use and risk reduction. Physicians can provide ongoing stimulus to enhance engagement, adherence and long-term retention in care (here defined as adherence to ART and clinical care [11–16]). Patient-centered care has been linked to adherence to treatment, self-management of chronic disease and clinical outcomes in HIV [17] including retention. Improved communication can enhance long-term retention, persistence and adherence, yet physicians may receive only limited training in communication and, once established, communication styles can be difficult to influence [18]. Development of patient-centered communication skills requires supervision [19], but physicians rarely receive feedback after medical school. Thus, MI is more often used by therapists or health care staff (e.g., psychologists, counselors, nurses) than physicians (e.g., [20–22]), despite academic literature supporting the utility of MI for HIV-infected patients to address or increase adherence and enhance communication (e.g., [23–28]). Utilizing MI, providers can encourage patients to recognize and overcome barriers to medication adherence and persistence and to take constructive actions, e.g., appointment attendance [29, 30]. Though used widely in addiction counseling in the US, MI use has been limited among physicians in Spanish-speaking populations (e.g., [31–33]). In addition, few studies have successfully intervened with HIV physicians to enhance physicians’ communication style and content while assessing its influence on patient outcomes [20, 24]. Similarly, few studies have successfully trained physicians to utilize MI to bring about health-behavior change to achieve biomedical outcomes such as viral suppression (e.g., asthma [34]), and other than our pilot study, we are unaware of any studies training physicians to enhance and sustain retention in challenging HIV-infected patients.

In our pilot study, MI was successfully utilized to enhance adherence and engagement in care among challenging patients in public and private health care settings in Buenos Aires, Argentina [35, 36]. Using a full factorial design, the relative benefit of patient and physician interventions in isolation and in combination was compared at 6 and 9 months’ follow-up. At 9 months, participants receiving care provided by a physician trained in MI achieved the highest proportion of viral suppression, reported significantly higher adherence and greater satisfaction with their provider relationship and treatment, as compared to those in the physician inactive condition, physician standard of care. Both public and private clinic patients had comparable improvements in adherence, and public and private physicians had comparable improvement in the use of MI-based communication methods. Results demonstrated that infectious disease physicians trained in MI re-engaged patients in care, i.e., enhanced and sustained patient adherence, viral suppression and improved patient-physician communication and treatment attitudes at 6 and 9 months post baseline. Our pilot study also found both patients and physicians preferred a collaborative approach to HIV treatment, one that relied on physician medical expertise and the patient’s own experiences [36]. In contrast, providing a group patient intervention increased, but did not sustain, adherence or viral suppression. Results supported the efficacy of a physician-based MI intervention, suggesting that physicians may be optimally positioned to provide ongoing MI-based consultation rather than “single dose” patient-based interventions.

### Aim of the study

This clinical trial (COPA2) proposes to test the impact of the MI intervention in a variety of clinical sites and regions to assess the generalizability and reach of the original findings from our pilot study, and to assess the potential for the physician-based MI intervention to maximize re-engagement and sustain retention, adherence and medication persistence among challenging patients in a broad variety of treatment settings. The current study extends these highly promising findings to six public and private clinics in four urban centers with high HIV prevalence, in which clinics (*n* = 6) are randomized to intervention (physician MI training) (*n* = 3) or standard of care (physician SOC) (*n* = 3) conditions in a 3:3 ratio. Using a cluster randomized clinical trial design, the study will test the effectiveness of MI skill training for physicians (*n* = 36) to re-engage and retain challenging patients (*n* = 420) and improve and sustain retention, adherence, persistence and viral suppression. The physician training in MI has been endorsed by the Argentina Society for Infectious Diseases and Panamerican Association of Infectious Diseases. As such, this study uses a sustainable model for MI training and supervision that can be implemented and disseminated to achieve the maximum public health impact to enhance re-engagement and sustain retention, viral suppression, persistence and adherence among “challenging” patients in HIV care.

## Methods/design

### Objectives

Study objectives are to (1) evaluate the effectiveness of a physician MI intervention to enhance long-term retention in care, medication adherence, medication persistence, and viral suppression among challenging patients and (2) determine patient- and physician-level factors impacting patient response to the MI intervention.

### Setting

In Argentina, and in much of Latin America, data on retention in care may be unreliable [37]. Earlier studies reported HIV prevalence among sexually transmitted diseases clinic attendees to be 20.4%, and 6.9% at outpatient clinics [38]. It was estimated that less than 50.0% of patients in Argentina knew their serostatus [39, 40]. Estimates have predicted that 22.7% [41] to 35.4% [39] of patients have AIDS at the time of diagnosis, that 11.9% of those unaware of their HIV status require treatment, and that mortality increases dramatically if patients are not engaged in care within 5 years of infection [39]. Lastly, structural challenges in over-saturated hospitals have been reported, e.g., long waiting periods to ART initiation caused by legal, financial and laboratory challenges, challenging continuity in care [42]. Late initiation of ART (> 6 months post referral) is problematic in many regions of the world [43], including South America.

In Argentina, 31% of men and 23% of women entered care late [5]; in Chile, 80% of patients; and in Peru, 40% [4, 37]. Many HIV-infected patients in Argentina have reported that they are unsure of the potential impact of non-adherence [42, 44], and expressed concern with antiretroviral (ARV) side effects [42] and their impact on social relationships [44, 45]. Feelings of ambivalence, weariness and anxiety about ART often lead to non-adherence and medication discontinuation [42]. Physicians have expressed frustration with challenging patients and our pilot indicated that clinical interventions are primarily paternalistic [44, 46]. Studies [35, 42] illustrate that increasing engagement in care increases retention and activates patients’ involvement in treatment through the formation of a therapeutic alliance with physicians.

### Design

This clinical trial utilizes a cluster randomized design to be conducted over 4 years, testing the effectiveness and long-term sustainability of MI clinical skill training to improve retention, re-engagement, adherence and viral suppression among challenging patients (Additional file 1). Clinics (*n* = 6) were randomized to intervention (physician MI training) (*n* = 3) or control (physician Standard of Care training) (*n* = 3) conditions in a 3:3 ratio. Within each clinic, six physicians (*n* = 36 total providers) and 60 patients not retained in care (with an additional 30 transgender patients at two sites) (*n* = 420 total patients) will be enrolled. (N.B.: after the study started, the investigators were awarded a supplement to the parent grant to increase the sample size and to include 60 transgender women, given the lack of information on this population and the potential differences in study outcomes. Some aspects of the supplement, such as targeted recruitment methods and separate analytic techniques, differ from the main protocol. This article focuses on the main protocol).

#### Physicians

Thirty-six physicians were recruited and trained (three training workshops/presentations conducted over 2 years), and will be assessed semiannually for 2 years (five assessments) at six sites, six physicians per site. Assessments address professional and general demographics (e.g., clinical experience, training, age), along with standard of care practices. Training is either on (1) Motivational Interviewing (intervention) or (2) Optimizing Entry, Retention and Adherence (control). All physicians complete audio/video recordings of one patient consultation with a COPA2-enrolled patient semiannually (five recordings). Physicians receiving Motivational Interviewing training receive online video supervision on MI implementation (coaching calls with an MI trainer) as reviewed in their videos at 3 months post MI training, 3 months-post advanced MI training, and at 12 months post baseline (approximately 30 min per supervision session). For this reason, each physician in the intervention group records one additional video after the first MI training workshop to enable them to receive feedback on their MI implementation.

#### Patients

Four hundred and twenty patients will be recruited and assessed for 2 years (five assessments), from six sites, 60 patients per site, with two sites including an additional 30 transgender patients each. Assessments will address patient demographic characteristics, adherence, depression, self-efficacy, communication with physician, and drug and alcohol use. Participants will provide two hair samples, at the 12-month and 24-month assessments. If viral load is not available through clinic records, patients will be asked to provide a blood sample for viral load assessment. A subset of participants will participate in audio/video recording of a consultation with their physician (who is also participating in the study) for MI supervision of physicians; patients at each site are randomized to be selected for audio/video recording. All patient participants will provide consent for medical record abstraction to assess clinic/laboratory visits, viral load results, and pharmacy fills.

Clinic randomization was conducted by generating a list of random numbers assigning clinics to condition. Clinics were assigned to numbers by staff not associated with the study; the US statistician was blind to clinic numbering and conducted randomization by number at study onset. Clinics were randomized to offer physicians (*n* = 36) either (1) the intervention condition: three training sessions in MI or (2) the control condition: three time-matched video-delivered sessions on Entry to Care, Retention and Adherence. Both conditions were time matched and all participants attend a total of five assessments (baseline, 6, 12, 18 and 24 months post intervention). Patients participating in this study (*n* = 420) within each clinic (*n* = 60 per clinic, with an additional 30 transgender participants per clinic at two clinic sites) are randomly assigned to be audio/video recorded at a regularly occurring consultation with their physician (who is participating in the study). Patients and physicians are assessed at 6-month intervals for 24 months, enabling a two-group (MI Intervention, Control SOC) × five timepoints (Baseline, 6 months, 12 months, 18 months, 24 months) comparison.

### Study hypotheses

#### Hypotheses 1.1–1.3

A higher proportion of patients from clinics offering the physician MI intervention will be retained in care, maintain optimal medication adherence, and achieve viral suppression at 6, 12, 18, and 24 months post baseline, as compared to those from clinics offering the control condition standard of care.

#### Hypothesis 1.4

Patients from clinics offering the physician MI intervention will be more likely to maintain medication persistence throughout the 2-year study period as compared to those from clinics offering the control condition standard of care.

#### Hypothesis 2.1–2.3

Within the MI condition, increased implementation of MI strategies by physicians will be associated with increased likelihood of their patients being retained in care, maintaining optimal medication adherence, and achieving an undetectable viral load.

#### Hypothesis 2.4

Within the MI condition, greater self-efficacy, motivation, and higher satisfaction with the patient-physician relationship will be associated with increased likelihood of being retained in care, maintaining medication adherence, and achieving an undetectable viral load.

### Principles for recruitment

#### Inclusion criteria

The study will increase the reach of the pilot study and increase its generalizability, expanding the patient population to public and private clinic and hospital patients, including transgender women, drug users, men who have sex with men (MSM), and heterosexual men and women. All eligible participants (patients, physicians) will be enrolled and assessed over 24 months.

##### Patients

Eligible individuals will be ≥ 18 years of age and “challenging” HIV-infected patients, (1) diagnosed for > 6 months and having a recent (i.e., ≤ 3 months) detectable viral load > 500 copies/mL following 6 months of ART prescription and (2) not retained in care, i.e., three missed pharmacy pick-ups in the last six consecutive months, or not attending a physician visit in the last 12 months or more. Patient participants will be recruited within participating clinics; clinic records will be reviewed to identify potential candidates. There are no exclusions based on literacy, as all materials will be administered using an audio computer-assisted self-interview system (ACASI) supervised by assessors.

This study will recruit HIV-infected men, women and transgender women, targeting subgroups of heterosexual women, men who have sex with men, drug users, sex workers, and transgender women, and representative of the ethnic subgroups. This study will collaborate with the Association of Transvestites, Transsexuals, and Transgender of Argentina (ATTTA) to enhance recruitment of transgender women. Patient participants will be men, women and transgender persons recruited from four urban areas in Argentina: Buenos Aires city, Rosario, Cordoba and Neuquén. The racial/ethnic demographic of this region is diverse, but primarily Caucasian with a small percentage of Indigenous peoples. Therefore, this study will enroll individuals of all ethnicities. The inclusion of women is an integral component of the study. Women comprised almost half of the HIV-infected participants at the clinical sites and this study will reflect a similar demographic. The study will recruit individuals of less than 21 years of age and the minimum age for inclusion is 18 years of age. (Patients aged under 18 years will not be included as Argentine law requires that HIV+ individuals aged under 18 years are under parental supervision and World Health Organization (WHO) guidelines require differing doses of medication for those aged under 18 years). Contacting patients and getting them into the clinic is the first step in re-engagement; pilot study results illustrated a spike in adherence following recruitment and enrollment among control condition participants, which was not sustained over time in the control condition. In both conditions, study retention will be also enhanced by compensation for assessments and coordination with physician/laboratory appointments.

##### Physicians

Eligible physicians will be infectious disease physicians drawn from participating sites, *n* = 6 per site. Each site had a minimum of six physician candidates available to enroll; participating clinics have an average of 6–10 physicians on staff. While we do not anticipate dropout (Argentine physicians are less likely to leave or change clinics), additional physicians are available to participate and staff members will track physicians moving to new sites who wish to continue to participate.

### Recruitment

Patient participants (*n* = 60 per site, with an additional 30 transgender participants per site at two clinics) will be recruited from six clinic sites, both private and public clinics. These clinics serve representative populations of HIV patient groups (e.g., MSM, intravenous drug users (IDU), heterosexuals, transgender, sex workers) and report ongoing HIV screening and diagnosis of new patients each week, and significant numbers of patients lost to care each year, ranging from 5 to 35%, representing an adequate patient sample for the study. Study recruiters at all sites were clinic counselors and administrative staff; recruiters were requested to identify patients who were not attending regular consultations in the last year (more than 3 months since the last expected visit). Each clinic will contact patients to return to the clinic, and returning patients will be invited to participate. Patients at participating clinics will be informed about the study by the study recruiters and those interested in enrollment will be referred to study staff. Persons contacting the outreach workers (who will be available in the clinics) on their own initiative will be asked for minimal demographic information to determine whether the patient meets basic inclusion and exclusion criteria. Those who meet these criteria will be given a detailed description of the study procedures, including time requirements and procedures to maintain confidentiality and will be scheduled for an orientation session, at which time they will be further informed about the study and asked to provide signed Informed Consent. Each qualified participant will then be scheduled for a baseline assessment (baseline questionnaires, and, if not available from medical records, a blood sample for viral load assessment). Given the time demands of this study and the personal nature of disclosures required we will be offering each participant monetary compensation for sessions (approximately US$10 per session). Study staff will conduct recruitment at the sites and will provide ongoing oversight of participant retention using patient locator data and rapid follow-up for missed appointments.

Physicians at clinics will be briefed on the study in a presentation by the coinvestigators, and invited to participate. Those interested in participating will be referred to on-site study staff and be given a detailed description of the study procedures, including time requirements and procedures to maintain confidentiality.

We have found that retention of patient participants who may be progressing in disease status presents special problems for which alternative procedures are required to maximize retention. As noted, we will compensate participants for their time, and will coordinate assessments with physician visits or pharmacy visits when possible. Our previous study indicated that increased retention is possible through concerted follow-up contacts and involvement of study staff. We propose to train staff to maintain continuing contact with study participants throughout the 24-month period of the study. Tracking methods will include collecting detailed locator information at baseline (e.g., address, two contact persons, home landmarks, workplace) as well as maintaining contact currency by updating and verifying locator information and telephone contacts at each subsequent visit. The staff members will make regular attempts to locate missing participants over a 2-week period following failure to present for an appointment. If, over a period of 2 weeks of continued attempts, we are unable to reach a participant for scheduling an assessment, we will seek to complete the assessment over the phone if possible or visit the contact person(s) that participants provided at study entry to learn of the whereabouts of the participant, or send a telegram. The study staff will be responsible for coordinating follow up and ensure confidentiality is maintained when making contacts in the community.

We have found that clinics may experience staff turnover in a 4-year period. To remain enrolled in the study, physicians must be seeing patients at the participating clinic. Participating physicians will have regular contact (quarterly) with trainers and other participating physicians. Staff members will maintain contact information to facilitate tracking, should physicians fail to present for scheduled appointments. (N.B.: in Argentina, physicians rotating/transferring to alternate facilities while participating in studies have the option to continue to provide care to study participants for the duration of the study. Alternatively, physicians may choose to discontinue their participation when transferring. In such cases, an additional physician will be recruited at the site if possible, and provided with training.)

### Site selection

To obtain sufficient numbers of challenging patients from the general patient population, participants (*n* = 60 per site, with an additional 30 transgender participants per site at two clinics) will be recruited from six public and private clinic sites. Most HIV-infected persons (83%) live in the province of Buenos Aires, Buenos Aires city, and within the provinces of Córdoba, Santa Fe and Mendoza; the epidemic is specifically urban. In 2015, the COPA team conducted a survey of 16 clinic and hospital sites in four urban centers with the highest HIV prevalence, Buenos Aires, Rosario, Cordoba and Neuquén, to assess willingness and interest in participating in the clinical trial. Of the 16 sites surveyed, 10 sites met the eligibility criteria for participation in the clinical trial; (1) willing to participate, (2) adequate HIV-patient load, (3) adequate numbers of patients meeting lost to follow-up criteria (see “Principles for recruitment – Patients” above) each year, (4) adequate numbers of interested infectious disease physicians (minimum of six per site). Sites selected expressed strong support for the implementation and objectives of the clinical trial. Clinics serve representative populations of HIV patient groups (e.g., MSM, IDU, transgender, women) and report ongoing HIV screening and diagnosis of new patients weekly at each site, representing an adequate patient population for the study. Clinic staff at all clinics include retention specialists who will track patients lost to care, and all clinics have similar retention strategies, including surveillance to contact patients who miss appointments, assessment of psychosocial needs and provision of referrals, updating patient contact information, and flexible hours. Participating clinics have an average of 6–10 physicians on staff and at least six of these physicians were interested in participating, representing adequate numbers of physicians available for study participation.

### Randomization

Cluster randomization at the clinic level is utilized to reduce the potential for contamination within clinics, i.e., exposing control patients to providers who received MI training, accommodating for the “natural” clustering of patients within physicians. (N.B.: as it is anticipated that some COPA2 patients will occasionally be seen by non-participating physicians not trained in MI, the study will track whether patients are seen by non-participating physicians. However, in order to comply with the protocol, each patient will be scheduled to see a COPA2 physician at least once every 6 months). Clinic randomization was conducted by generating a list of random numbers assigning clinics to condition. Both public and private clinics are included as representative of the Argentine health care system; clinics were matched by public/private and HIV census prior to randomization.

### Intervention

#### Motivational Interviewing (MI) – theoretical model

The original MI model arose from the client-centered therapy skill of “accurate empathy” [47], and focused on responding differentially to client speech, within an empathic client-centered style. MI emphasizes two components: a “relational component” that relies on empathy and the “interpersonal spirit of MI” (collaboration between patient and physician) and a “technical component” that involves the evocation and reinforcement of patient “change talk” (communication indicative of consideration of behavior change). MI is best described as targeting ambivalence regarding health-behavior change, in this context, adherence to clinical care and medication regimens. In addressing ambivalence, the physician evokes and strengthens the patient’s verbalized motivation to change (“change talk”) to develop a plan and commitment to change. In doing so, self-efficacy and motivation to change are increased, resulting in enhanced health behavior, i.e., treatment adherence and medication persistence, and ultimately resulting in enhanced health outcomes, i.e., viral suppression. The provider seeks to have the patient, rather than the provider, voice their own arguments for positive change [48], e.g., reasons to increase their engagement in care.

#### Training physicians and health care staff (nurses, reception staff, peer health navigators)

The intervention training utilizes a structured, sustainable MI training and supervision program designed to improve retention, adherence and persistence in challenging patients. While there is some controversy over the “best” MI training strategy (e.g., [49]), the current MI training applies recommendations from our feasibility study, recommendations from previous studies (e.g., [23, 49]) and guidance from our expert consultant, Dr. Carolina Yahne, for (1) sensitization training for front-line staff on the clinic as a motivational setting, (2) increased physician training time to four half days over 2 weeks at baseline, 6 (advanced) and 12 months (refresher), (3) long-distance Skype supervision for physicians at each site to be conducted 3 months post baseline, 6 and 12 months’ training using coded video recordings of clinical consultations, (4) establishment of sustainable training for local physicians providing HIV healthcare and sustainable local supervisors/supervision for physicians. While our pilot study did not experience problems in physician retention, yearly training opportunities will be provided for new physicians replacing those who may leave the study. All training, supervision and implementation will be led by MI trainers, with support from the senior US investigators. Dr. Yahne has co-authored numerous articles and textbooks with Dr. William Miller and others on Motivational Interviewing as a clinical strategy in both English and Spanish, and co-produced video training materials in English and Spanish. Dr. Yahne has trained clinicians and providers in English and Spanish in the US, Central and South America, and is the founder of the Motivational Interviewing Network of Trainers (MINT). This study focuses on the providers with the greatest referent power in Argentina, physicians, while providing sensitization focus group training for front-line staff (reception, counselors, nursing) focusing on the clinic environment as a motivational setting.

#### MI physician intervention

Intensive training will take place three times in the first 2 years and utilize *Motivational Interviewing, 3rd Ed. Spanish* [50]*;* training interventions will be delivered in four half-day sessions over 2 weeks. The Provider Training Intervention Manual and accompanying slide presentations were developed collaboratively by the COPA team with input from Dr. Yahne. The training elements will address the basic MI skills and elements identified as most effective [23], MI spirit (collaboration, evoking patient motivation, honoring patient autonomy [48]), recognizing and reinforcing change talk, and “rolling” with (not fighting) resistance (MI consistency). Physicians utilizing MI will learn to engage with patients in an empathic, nonjudgmental manner and to pose simple but strategic questions to motivate change; when patients resist change, the physician learns to “roll” with resistance instead of confronting it. If and when the patient is ready to initiate a change, the physician will be prepared to support their decision [36]. Training will utilize baseline and follow-up patient-physician videotapes to enable physicians to individually review their own patient interactions independently, focusing on MI, communication strategies and opportunities to respond more accurately to patient communication. Advanced and refresher training after 6 and 12 months will utilize the same training structure and providing additional supervision and feedback.

#### MI supervision

Training will be followed by individual supervision and feedback for physicians using audio/video recordings and Skype consultations to promote fidelity to MI. Supervision will be provided by MI trainers on Skype at 3 months post MI training, 3 months post advanced MI training and at 12 months, approximately 30 min per supervision session. During the training, Argentine trainers (Drs. Lucas and Bordatto) and the US trainer (Dr. Bofill) from our pilot study will be trained by MI trainers to develop a sustainable training and supervision program. (N.B. Should a physician withdraw from the study, the sustainable training and supervision model will enable COPA2 to recruit, train and supervise additional physicians wishing to participate in the study].

#### Standard of care (SOC) condition

SOC site physicians will attend three time-matched video presentations over 2 years on research on optimizing entry into and retention in care and adherence, from training materials available from Fundación Huésped.

### Assessment measures

**Fig. 1:**
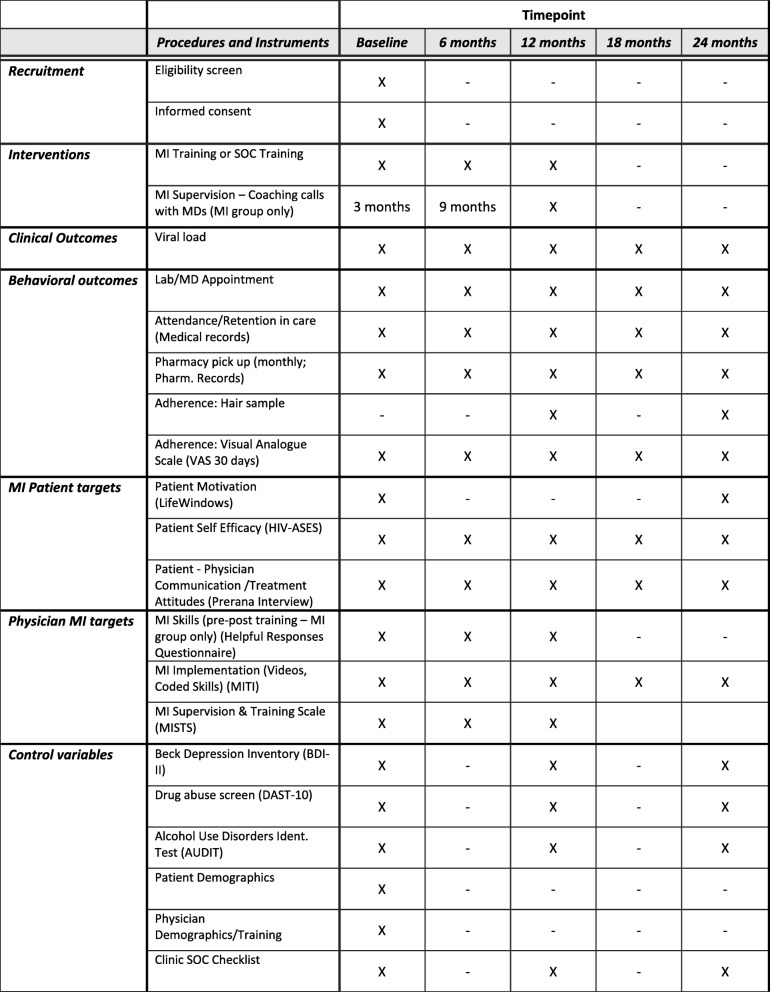
Schedule for enrollment, interventions, and assessments

Patient and Physician Measures were selected from the pilot study, based on patient, physician and clinic characteristics relevant to study outcomes. Measures were in Spanish and adapted to the local context during the pilot study or were used for general reference, adapted, translated, back translated and reviewed for fit with cultural and local context. Assessments are administered via audio computer-assisted self-interview (ACASI) to minimize the influence of social desirability and improve data quality. Study staff introduce the ACASI system to ensure participant competence and are available for assistance during the assessment. Patient assessments at baseline and 24 months are approximately 60 min; physician ACASI assessments are approximately 20 min. Figure 1 illustrates the schedule for enrollment, interventions, and assessments. All sites schedule clinical appointments quarterly (3-monthly) for patients failing treatment (not engaged, detectable viral load, calendar year 1); after 1 year and with undetectable viral load, patients are scheduled biannually (6-monthly, calendar year 2). Pharmacy pick-up is scheduled monthly for all patients; those with long-term, undetectable viral load can arrange for bimonthly pick up only with special permission.

#### Clinical outcomes: baseline, semiannual assessment for 2 years (five assessments)

HIV Viral Load (VL): patients should have been prescribed 6 months of ART and have a recent (i.e., ≤ 3 months) detectable VL > 500 copies/mL as eligibility criteria. HIV VL are performed at least every 6 months on all patients as standard of care; data will be abstracted from medical records; if not available, VL will be assessed by blood sampling. Viral suppression (HIV VL < 200 copies/mL) will be the primary dichotomous outcome; a secondary dichotomization will be made among detectable patients with VL < 1000 and those with VL > 1000 [51, 52]).

#### Behavioral outcomes: baseline, semiannual assessment for 2 years (five assessments)

Outcomes are retention in care, medication persistence, and ART adherence. In this study, retention in care will be defined as the number of missed clinic appointments (i.e., “no shows” not canceled in advance by patients or clinic staff) in the 6 months preceding assessment, including that timepoint’s visit with the COPA2 physician. The primary retention analysis will utilize a dichotomized indicator 0/1 of any missed visits vs. no missed visits, and the missed appointment count will also be used to create a continuous appointment adherence percentage variable (number of completed visits/number scheduled [53, 54]). All patients will have at least one scheduled appointment per 6 months so the 0/1 variable will be valid for all patients. The proportion of kept appointments includes the total number as the denominator; thus, it corrects for inflated numbers of missed appointments. Medication persistence will be measured by the time to treatment discontinuation, with a permissible gap of < 30 days [3]; participants will be considered to have discontinued the regimen if a therapy gap ≥ 30 days occurs. This will be measured by monthly pharmacy pick-ups; failure to obtain a month’s medication will indicate a 30-day gap in therapy. Measures are adapted from the HIV/AIDS Bureau Performance Measures guidelines [55] obtained by medical record abstraction. Adherence will be measured by hair sample, pharmacy pick-ups (monthly), and self-report. Hair sample will estimate an average concentration of ARV medication exposure over 6 weeks with cut-off values used as categorical variables (12 months and 24 months) [56, 57]. Self-report will reflect percentage adherent over the last 30 days (Visual Analog Scale; VAS); responses are integer values from 0 to 10, which represent percentage of doses taken (0–100) when multiplied by 10.

#### MI patient targets: baseline, semiannual assessment for 2 years (five assessments)

##### Self-efficacy

The HIV Treatment Adherence Self-Efficacy Scale (HIV-ASES [58]) will assess the patients’ perceived self-efficacy in being adherent.

##### Motivation for adherence

Motivation for adherence will be measured using the LifeWindows Information–Motivation–Behavioral Skills Adherence Assessment Questionnaire [59], a 10-item measure on motivation to be adherent. Items use a 5-point range - *strongly disagree* to *strongly agree*.

##### Patient-physician communication/treatment

The Prerana Interview [60] will be used to assess the patient-provider relationship and attitudes towards treatment. Items include psychosocial barriers to patient-provider communication and clinic appointment attendance and attitudes regarding treatment.

#### MI physician targets: baseline, semiannual assessment for 2 years (five assessments)

##### MI pre-post workshop skills

The Helpful Responses Questionnaire [61] is a qualitative tool used to assess MI skills and will be administered pre and post MI workshop training.

##### MI implementation skills

Audio/video recording review, Motivational Interviewing Treatment Integrity (MITI) Version 2.0 [62] coding system of videotaped interviews every 6 months (five recordings) with COPA2-enrolled patients. Patients will be participants randomized at a regular visit for audio/video recording; for each physician, at each timepoint, the patient they are to record the video with will be randomly selected from their assigned COPA2 patients. Each patient will not be selected for more than one video; therefore, this will comprise a representative sample of the patient participants.

##### Motivational Interviewing Supervision and Training Scale (MISTS)

This is a physician/therapist checklist of quantity and quality of each MI strategy, completed by the physician following the session, and reviewed by the trainer.

#### Control variables: baseline and semiannual assessment for 2 years (five assessments)

##### Depression

The Beck Depression Inventory-II (BDI-II [63], Spanish) measures somatic and non-somatic symptoms of depression separately to distinguish the manifestation of depression from symptoms of HIV, which may resemble depression in some cultures.

##### Drug abuse

The Drug Abuse Screening Test-10 (DAST-10 [64], adapted, Spanish) will assess the risk of abuse of illegal or prescription drugs and is a 10-item measure assessing the use of drugs, excluding alcohol and tobacco, which is responded using a dichotomous (yes/no) response.

##### Alcohol use

The Alcohol Use Disorders Identification Test (AUDIT [65], Spanish) is a 10-item assessment of indices of alcohol consumption (i.e., frequency, binge drinking, alcohol-related problems).

##### Patient demographics

Assessment includes gender identity, age, income, housing, education and HIV history, e.g., time since diagnosis, time on ART, past HIV care, barriers to care.

##### Physician demographics

Assessment includes clinical experience, training, and age.

##### Clinic characteristics

Standard of care practice at each clinic [66] is evaluated as a checklist completed by the physician participants. The scores from each site’s physicians will be pooled to yield an average score from each clinic.

### Consortium, staff training and quality assurance

The University of Miami and Argentina investigators have bi-weekly conference calls to review all aspects of study implementation including the recruitment, enrollment, assessments, training, quality control and conformity to the project timeline. The PI provides overall leadership and ensure fidelity to protocol and the Argentina investigators provide direct oversight and guidance for all phases of implementation at their sites. Training manuals for the interventions were developed by the US and Argentina teams in English, translated into Spanish and back-translated into English for fidelity. All new translations (e.g., informed consents) were conducted under the supervision of the Argentina PIs. Staff training for assessments and the MI intervention were conducted on site by Drs. Jones, Bofill and Weiss and Argentina MI trainers. Team members from the Fundación Huésped site will receive guidance in the use of hair-sampling analysis under the supervision of Dr. Gandhi at UCSF. Ongoing quality assurance to ensure fidelity of the intervention to the study protocol is provided by the Argentina PIs and project coordinator; a sample of 10% of audio/video-recorded MI-patient sessions are reviewed by Dr. Bofill. Data managers from both the USA and Argentina teams will review physical health and pharmacy pick-up data, which will be obtained from medical records and entered into REDCap (Research Electronic Data Capture) online databases [67] (see also “Assessment measures”).

### Dissemination and implementation

Study results will be disseminated to regional sites by the study team. Dr. Cahn, co-investigator from Fundación Huésped, is past President of the International AIDS Society, and will, together with Drs. Sued and Cassetti, lead the dissemination of the study results. If successful, the Society of Infectious Disease and the Panamerican Association of Infectious Diseases have provided strong endorsement for a training program for infectious disease physicians and the training team will collaborate on dissemination and implementation.

### Analysis approach

Prior to primary and secondary analyses, potential demographic confounders will be tested for association with outcome variables and for imbalance between groups. If significant, these variables will be controlled in the final models. All analyses will include random terms to account for repeated measures as well as the nesting of patients within providers and the clinic-randomized design. Random-effects models [68] will be used to incorporate the nested data structure (the nesting of patients within providers). This study will use an intention-to-treat analysis. Due to the relatively small number of randomized clusters, experimental and control groups will be carefully examined for imbalance on key covariates prior to analyses. Heterogeneity between clinics and clinicians will be described by random effects in the analyses. Potential covariates include clinic standard of care, patient and physician demographics (e.g., income, training), patient depression and substance use. To adjust for inflated type I errors due to multiple comparisons, Bonferroni corrections [69] will be used for *p* < .05. For analyses that involve viral loads, it is expected that there will be some missing cases of this key outcome variable. We expect that the majority of missing viral load results will be due to the participant missing a clinic visit. To control for effects of potential variables that systematically influence missing cases, the dichotomous retention variable (no missed visits vs. at least one missed visit) will be included as a covariate in the hypothesized model; all coefficients will be estimated. In addition, missing data patterns will be analyzed for viral loads using Little’s missing completely at random (MCAR) test [70]. If the *p* value for the MCAR is not significant, then missingness is assumed to be missing at random (MAR [70]), which allows for the use of full-information maximum likelihood (FIML) estimation [71] to handle missing cases.

#### Hypotheses 1.1–1.3

A higher proportion of patients from clinics offering the physician MI intervention will be retained in care, maintain optimal medication adherence, and achieve viral suppression at 6, 12, 18, and 24 months post baseline, as compared to those from clinics offering the standard of care.

#### Hypothesis 1.4

Patients from clinics offering the physician MI intervention will be more likely to maintain medication persistence throughout the 2-year study period as compared to those from clinics offering the standard of care.

#### Analytic strategy 1.1–1.3

To evaluate hypotheses 1.1–1.3, two analyses will be conducted for each outcome. The first “unadjusted” analysis will include time, randomized condition, and the interaction between time and condition as predictors, and conditions will be compared at each timepoint. The interaction between time and condition will be examined due to the results of the pilot study; initial improvements were present in both the control and experimental groups, but they were not sustained over time in the control group. In addition to these primary predictors, the second “adjusted” analysis will include time-varying measurements of self-efficacy, depression and substance use as covariates. To incorporate repeated binary outcomes (retention in care and viral suppression) over five timepoints (baseline, 6, 12, 18, and 24 months), generalized linear mixed models [72] will be used. Hypotheses 1.1 and 1.3 will be evaluated utilizing repeated measures logistic regression models. The proportion of patients retained in care (i.e., those having no missed clinic appointments in the previous 6 months) and those achieving an undetectable viral load will be compared between the experimental and control groups at 6, 12, 18, and 24 months post baseline using the fitted model. Hypothesis 1.2 will utilize a similar analytic strategy, except it will use a linear mixed model [68] to examine percentage adherence in the past 30 days as a continuous outcome. Furthermore, to estimate unbiased parameters, we will check the normality assumption for this adherence outcome by using skewness and kurtosis (acceptable range = ± 2) [73]. If the variable will not hold the normality assumption, the outcome will be a logarithm.

#### Analytic strategy 1.4

Hypothesis 1.4 will be evaluated using a frailty model, which is an extension of the Cox regression model to include random effects. Time to discontinuation of medication regimen (in months) will be the outcome of interest, and the hazard ratio for discontinuation will be computed for the experimental vs. the control condition. Additional analyses will adjust for time-varying covariates as above.

#### Hypotheses 2.1–2.3

Within the MI condition, increased implementation of MI strategies by physicians will be associated with increased likelihood of their patients being retained in care, maintaining optimal medication adherence, and achieving an undetectable viral load.

#### Hypothesis 2.4

Within the MI condition, greater self-efficacy, motivation and higher satisfaction with the patient-physician relationship will be associated with increased likelihood of being retained in care, maintaining medication adherence, and achieving an undetectable viral load.

#### Analytic strategy 2.1–2.4

Hypotheses 2.1–2.4 will be evaluated utilizing unadjusted and adjusted analyses, similarly to hypotheses 1.1–1.4. For each outcome, the first analysis will include only the primary predictors, and the second will adjust for psychosocial covariates. Hypotheses 2.1–2.3 will be analyzed using similar strategies as hypotheses 1.1–1.3 but restricted to patients in the MI group. The primary predictors of interest in each analysis will be time (6, 12, 18, and 24 months), physician implementation of MI (a count variable indicating the number of times MI strategies were used during videotaped patient encounters in the 6 months preceding assessment), and the interaction between implementation and time. The *F* statistic for the type III test of the interaction term will be examined; if significant, slopes will be estimated between implementation and outcome variables at each timepoint. If the *F* statistic is not significant, the model will be refit including only main effects of time and implementation, and the slope for implementation will be evaluated (testing a relationship between implementation and outcomes that is not time-dependent). Hypothesis 2.4 will be evaluated in a similar manner as hypotheses 2.1–2.3; the primary predictors of interest will be time, self-efficacy, motivation and patient-provider relationship. A model including a full-factorial combination of all predictors will be fit and reduced to a final model utilizing an appropriate model selection strategy (e.g., backwards elimination). Two exploratory analyses will also be conducted: the first will explore how successfully the implementation of MI is sustained over time by utilizing a Poisson regression with implementation as the count outcome and time as the predictor of interest. The second will be a series of analyses similar to those utilized for hypotheses 2.1–2.3 including physician characteristics (e.g., years of training) as predictors of patient outcomes. In order to determine which components of the intervention were most effective in promoting positive patient outcomes, the final set of analyses will examine skills targeted by the MI intervention (both patient-level, e.g., motivation and self-efficacy and provider level, e.g., implementation of MI) as potential mediators of patient outcomes. Data will be incorporated into time-lagged path models in which we will test the hypothesis that exposure to the MI training/exposure to physicians trained in MI will result in increases in these skills over time, which will be related to subsequent improvements in patient outcomes (e.g., higher rates of viral suppression) (Table 1).

**Table 1:** Summary of hypotheses and analyses

Hypothesis number	Outcome variables	Primary predictor variables	Hypothesis description	Data sources	Analytical models
Hypothesis 1.1	Retention in care (no missed clinic visits vs. at least 1 missed clinic visit)	Condition (MI vs. SOC)	At each follow-up timepoint, the MI condition clinics will have a higher proportion of patients retained in care, as compared to those from the SOC condition clinics	Clinic/medical records	Repeated measures logistic regression models (1) “Unadjusted” analysis: predictors include time, condition, and interaction between time and condition (2) “Adjusted” analysis: includes predictors from unadjusted analysis, *plus* time-varying measurements of self-efficacy, depression, and substance use as covariates
Hypothesis 1.2	Medication adherence (percentage adherence in the last 30 days)	Condition (MI vs. SOC)	At each follow-up timepoint, patients from the MI condition clinics will have higher medication adherence, as compared to those from the SOC condition clinics	Adherence self-report (Visual Analogue Scale)	Linear mixed models (1) “Unadjusted” analysis: predictors include time, condition, and interaction between time and condition (2) “Adjusted” analysis: includes predictors from unadjusted analysis, *plus* time-varying measurements of self-efficacy, depression, and substance use as covariates
Hypothesis 1.3	Viral suppression	Condition (MI vs. SOC)	At each follow-up timepoint, the MI condition clinics will have a higher proportion of virally suppressed patients, as compared to those from the SOC condition clinics	Viral load (medical records)	Repeated measures logistic regression models (1) “Unadjusted” analysis: predictors include time, condition, and interaction between time and condition (2) “Adjusted” analysis: includes predictors from unadjusted analysis, *plus* time-varying measurements of self-efficacy, depression, and substance use as covariates
Hypothesis 1.4	Medication persistence (time to treatment discontinuation – in months)	Condition (MI vs. SOC)	Throughout the 24-month study period, patients from the MI condition clinics will be more likely to maintain medication persistence, as compared to those from the SOC condition clinics	Pharmacy pickups (pharmacy records)	Frailty models (extension of the Cox regression model to include random effects) To investigate hazard functions of main outcomes by conditions, we will calculate the hazard ratio for discontinuation between the MI vs. SOC condition. (1) “Unadjusted” analysis: predictors include time, condition, and interaction between time and condition (2) “Adjusted” analysis: includes predictors from unadjusted analysis, *plus* time-varying measurements of self-efficacy, depression, and substance use as covariates
Hypothesis 2.1	Retention in care (no missed clinic visits vs. at least 1 missed clinic visit)	Physician implementation of MI strategies (count variable)	Within the MI condition, increased implementation of MI strategies by physicians will be associated with increased likelihood of their patients being retained in care	• Clinic/medical records • Patient visit videos (MITI coding)	Repeated measures logistic regression models (1) “Unadjusted” analysis: predictors include time, MI implementation, and interaction between time and MI implementation (2) “Adjusted” analysis: includes predictors from unadjusted analysis, *plus* psychosocial covariates
Hypothesis 2.2	Medication adherence (percentage adherence in the last 30 days)	Physician implementation of MI strategies (count variable)	Within the MI condition, increased implementation of MI strategies by physicians will be associated with increased medication adherence by their patients	• Adherence self-report (Visual Analog Scale) • Patient visit videos (MITI coding)	Linear mixed models (1) “Unadjusted” analysis: predictors include time, MI implementation, and interaction between time and MI implementation (2) “Adjusted” analysis: includes predictors from unadjusted analysis, *plus* psychosocial covariates
Hypothesis 2.3	Viral suppression	Physician implementation of MI strategies (count variable)	Within the MI condition, increased implementation of MI strategies by physicians will be associated with increased likelihood of their patients achieving viral suppression	• Viral load (medical records) • Patient visit videos (MITI coding)	Repeated measures logistic regression models (1) “Unadjusted” analysis: predictors include time, MI implementation, and interaction between time and MI implementation (2) “Adjusted” analysis: includes predictors from unadjusted analysis, *plus* psychosocial covariates
Hypothesis 2.4	• Retention in care (no missed clinic visits vs. at least 1 missed clinic visit) • Medication adherence (percentage adherence in the last 30 days) • Viral suppression	• Self-efficacy • Motivation • Patient-provider relationship satisfaction	Within the MI condition, greater self-efficacy, motivation, and higher patient-physician relationship satisfaction will be associated with increased likelihood of being retained in care, maintaining medication adherence, and achieving viral suppression	• Clinic/medical records • Adherence self-report (Visual Analog Scale) • Viral load (medical records) • HIV-ASES • LifeWindows • Prerana Interview	Repeated measures logistic regression models (for binary outcomes) and linear mixed models (for continuous outcome) (1) “Unadjusted” analysis: predictors include time, self-efficacy, motivation, and patient-provider relationship (2) “Adjusted” analysis: includes predictors from unadjusted analysis, *plus* psychosocial covariates. To identify the optimal predictors in the hypothesized models, a model including a full-factorial combination of all predictors will be fit and reduced to a final optimal model utilizing an appropriate model selection strategy (e.g., backwards elimination)
Exploratory analysis 1	Physician implementation of MI strategies (count variable)	Time	Explore how successfully implementation of MI is sustained over time	Patient visit videos (MITI coding)	Poisson regression
Exploratory analyses 2.1–2.3	• Retention in care (no missed clinic visits vs. at least 1 missed clinic visit) • Medication adherence (percent adherence in the last 30 days) • Viral suppression	• Time • Patient-level skills (self-efficacy, motivation, patient-provider relationship) as potential mediators • Provider-level skills (MI implementation) as a potential mediator	Exposure to the MI training/exposure to physicians trained in MI will result in increases in patient-level and provider-level skills over time, which will be related to subsequent improvements in patient outcomes (i.e., higher rates of retention in care, greater adherence, higher rates of viral suppression).	• Clinic/medical records • Adherence self-report (Visual Analogue Scale) • Viral load (medical records) • HIV-ASES • LifeWindows • Prerana Interview • Patient visit videos (MITI coding)	Time-lagged path models Mediation model: examine skills targeted by the MI intervention (both patient-level and provider-level) as potential mediators of patient outcomes. 1. “Unadjusted” mediation analysis: predictors include primary predictor variables and physician characteristics 2. “Adjusted” mediation analysis: includes predictors from unadjusted analysis, *plus* psychosocial covariates

#### Power analysis

The effect size assumed in the power calculation is from the pilot data, which is the “gold standard” for power calculations; the calculation is done for the primary aim – viral suppression, as we sought to power for the most important outcome. The sample size for this study was determined according to data gathered by the COPA pilot; 68% of patients in the experimental group achieved viral suppression by 9-month follow-up as opposed to 44% of those in the control condition. Assuming a similar effect size will be observed in this larger trial and that the plausible range of viral suppression is 20 to 60% in the control group, a power analysis indicates that six clinics allocated in a 3:3 ratio with six providers and 60 patients per clinic (excluding transgender patients) will provide > 80% power to detect a difference between groups using a two-tailed test at *α* = .05. This analysis also assumes 60% of the variance will be between providers within clinics and 40% will be between clinics.

### Sources of material – data collection

Biological, psychosocial and behavioral data will be collected during the study via clinic medical records, computer assisted interviews and hair and/or blood sampling. Physical health and pharmacy pickup data will be obtained from clinic/pharmacy records and entered into REDCap, a secure web-based database application [67]. Hair sample (100 hairs) will be used to assess concentrations of antiretroviral medication at 12 months and 24 months. If no viral load sample is available within the previous 3 months, a blood sample (1 × 10 mL) will be collected to assess viral load; when available, existing VL samples drawn within an appropriate sampling frame will be used instead of additional blood draw. The standard of care at all clinics is VL assessment every 3 months for patients not virally suppressed or retained in care; study assessments will utilize existing VL when visits are aligned with study time points. Computer-assisted assessments will be overseen at the sites by trained study staff. Laboratory data will be collected by trained clinic phlebotomists. Data will be coded with ID numbers only, and linkage data will be stored separately for tracking. No unique identifiers will be maintained with the samples or together with the data; access to collected data will be restricted to study staff only. The following procedures will be used for biological materials: viral load testing will be conducted at each clinic. The interviewer will complete a form providing information on the participant (study number, date, name of test, interviewer); the patient will provide a blood sample and a copy of the request will be returned to the participant record in the respective study office. The original forms will be left with the phlebotomist and the blood sample and form will be transferred to the clinic laboratory for testing. Hair sample collection requires a pair of scissors used to collect ~ 100 hairs near the scalp, samples are wrapped in foil and stored at room temperature. Hair can be stored for long periods prior to analysis and will be shipped to the US lab without precautions for biohazardous materials. Demographic data and psychosocial data collected during the study via computer-assisted interviews will be uploaded to a secure cloud storage system. MI implementation data will be obtained from audio/videotaped consultations with patients, in which the physician is viewed and the patient is obscured from view and identification.

### Data safety and monitoring

A Data Safety Monitoring Board (DSMB) has been established to provide monitoring of the experimental arm at study midpoint to determine: (1) if there are any unexpected systematic variations in health status attributable to either study condition which may be negatively affecting the health of the participants; and (2) whether the intervention condition (or control) are demonstrating such clear-cut positive effects that continuation of the trial (i.e., withholding the treatment from the SOC control condition) would be considered unethical. Monitors will periodically review and evaluate the accumulated study data for participant safety, study conduct and progress, and efficacy, and make recommendations concerning the continuation, modification, or termination of the trial. The DSMB will consider study-specific data as well as relevant background knowledge about the disease or patient population under study. The DSMB defined event triggers that would call for an unscheduled review, stopping guidelines, unmasking (unblinding) and voting prior to initiating data review. The DSMB is also responsible for maintaining the confidentiality of its internal discussions and activities as well as the contents of reports provided to it. Prior to study onset, the DSMB will review the protocol for major concerns. The DSMB consists of three monitors who are independent expert investigators in HIV research not associated with the study.

During the trial of COPA2, the DSMB will review cumulative study data to evaluate safety, study conduct, and scientific validity and integrity of the trial. DSMB members must be satisfied that the timeliness, completeness, and accuracy of the data submitted to them for review are sufficient for evaluation of the safety and welfare of study participants. The DSMB should also assess the performance of overall study operations and any other relevant issues, as necessary.

### Adverse events

Given the behavioral nature of this study, adverse events are not anticipated, but a response plan has been developed, following institutional policy guidelines. In accordance with the plan, the PI will report all adverse events in writing to the University of Miami’s Institutional Review Board (IRB) within two working days of the PI learning of the event. Drs. Sued, Cahn and Cassetti, the co-PIs in Argentina, will also report adverse events in the same manner to the Argentina Ethics Committees. This same procedure is also applicable should the PI learn of a participant’s death during their enrollment.

Non-serious adverse events will be reported in writing to the UM IRB at the time of continuing or final review of the study protocol; the Argentina Ethics Committees associated with the site and the primary subcontractor will be informed of non-serious adverse events during their normal reporting cycles. Reporting of both serious and non-serious adverse events to the boards of UM and Argentina are carried out via standardized forms that detail the date, nature, relationship to research and result/status of each event.

## Discussion

This study addresses several priority topics of research: retention and engagement in HIV services, achievement of optimal prevention, and treatment responses among people who are living with HIV. Results from the pilot study identified the elements of the MI intervention, patient self-efficacy and motivation, and facilitating patient-provider partnerships and communication, which were associated with patient engagement and underlay the intervention outcome, viral suppression. Study results may have significant public health implications for the implementation of MI to re-engage and retain patients in HIV treatment and care and improve viral suppression through high levels of medication adherence. The study approach is designed to enhance dissemination and proposes to establish a sustainable MI training and supervision program for physicians which, if successful, can be implemented and disseminated in public and private health care settings, which are representative of the HIV healthcare delivery system in Argentina.

### Trial status

Interested physicians were identified prior to study start; physicians were officially recruited and enrolled between October 2016 to March 2017. Patient enrollment began November 2016 and is expected to be completed in April 2018. The trial is expected to end in May 2020.

## Additional file

Additional file 1: SPIRIT 2013 Checklist: recommended items to address in a clinical trial protocol and related documents. (DOC 123 kb)

## Abbreviations

ACASI: Audio computer-assisted self-interview
ART: Antiretroviral therapy
ARV: Antiretrovirals
ATTTA: Association of Transvestites, Transsexuals, and Transgender of Argentina
AUDIT: Alcohol Use Disorders Identification Test
BDI-II: Beck Depression Inventory-II
COPA: Conexiones y Opciones Positivas en la Argentina (Positive Connections in Argentina)
COPA2: Conexiones y Opciones Positivas en la Argentina 2 (Positive Connections in Argentina 2)
DAST-10: Drug Abuse Screening Test-10
DSMB: Data Safety Monitoring Board
FIML: Full-information maximum likelihood
HIV-ASES: HIV Treatment Adherence Self-Efficacy Scale
IDU: Intravenous drug users
IRB: Institutional Review Board
MAR: Missing at random
MCAR: Missing completely at random
MI: Motivational Interviewing
MINT: Motivational Interviewing Network of Trainers
MISTS: Motivational Interviewing Supervision and Training Scale
MITI: Motivational Interviewing Treatment Integrity
MSM: Men who have sex with men
REDCap: Research Electronic Data Capture
SOC: Standard of Care
VAS: Visual Analogue Scale
VL: Viral load
WHO: World Health Organization
